# The *ZcVg3* Gene Regulates the Reproduction and Lifespan of Female *Zeugodacus cucurbitae* (Coquillett) Mediated by Short-Term High Temperatures

**DOI:** 10.3390/insects15070499

**Published:** 2024-07-04

**Authors:** Shuyan Yang, Sihua Peng, Aqiang Wang, Jingjing Jia, Qianxing Wu, Xiaofeng Yang, Shihao Zhou

**Affiliations:** 1 Sanya Nanfan Research Institute of Hainan University, Sanya 572022, China; ysy000567@foxmail.com (S.Y.); pengsihua@126.com (S.P.); qianxing301@163.com (Q.W.); 994469@hainanu.edu.cn (X.Y.); 2 Key Laboratory of Plant Disease and Pest Control of Hainan Province/Institute of Plant Protection, Hainan Academy of Agricultural Sciences (Research Center of Quality Safety and Standards for Agricultural Products of Hainan Academy of Agricultural Sciences), Haikou 571199, China

**Keywords:** *Zeugodacus cucurbitae* (Coquillett), RNA interference, Vitellogenin-3, reproduction, lifespan, synergistic relationship

## Abstract

**Simple Summary:**

Exposure to short-term high temperatures of 45 °C can enhance the development and reproduction of *Zeugodacus cucurbitae* (Coquillett). In this study, the function of the *ZcVg3* gene in reproduction was studied by interfering with its expression via the injection of siRNA. The silencing of the *ZcVg3* gene could affect the expression of other related genes. It also resulted in a significant reduction in egg production, spawning days, ovarian development and lifespan. The results suggest that the *ZcVg3* gene plays a crucial role in pest reproduction under short-term high temperatures.

**Abstract:**

*Zeugodacus cucurbitae* (Coquillett) is a significant pest affecting fruit and vegetables in tropical and subtropical regions, and its development and reproduction are enhanced after exposure to short-term high-temperature stress at 45 °C. Vitellogenin (Vg) is an essential precursor of yolk protein formation in eggs and plays a vital role in the ovarian development of insects. Interfering with the *Z. cucurbitae vitellogenin receptor* (*ZcVgR*) gene in short-term high-temperature conditions decreases the fecundity of female adults, while the transcription level of the *ZcVg3* gene increases. To elucidate the reproductive function of the *ZcVg3* gene and the synergistic relationship among the *ZcVgs* genes under short-term high temperatures, this study injected siRNA to interfere with the *ZcVg3* gene after subjecting *Z. cucurbitae* to a 1 h treatment at 45 °C and 25 °C. The expression of the *ZcVg3* gene was suppressed, leading to the upregulation of the *ZcVg1* and *ZcVg2* genes, and the expression of the *ZcVgR* gene was initially decreased and then increased. Silencing the *ZcVg3* gene after a 1 h treatment at 45 °C resulted in a reduction of approximately 84.7% and 75.9% in the fecundity and spawning days of female adults compared to the control. The development rate of their ovaries and the ovarian diameter significantly decreased, and their lifespan was reduced by 71%. The *ZcVg3* gene plays a crucial role in the reproduction of *Z. cucurbitae* in short-term high-temperature conditions. The results of this study provide potential targets for the development of RNAi-based techniques for the control of *Z. cucurbitae*.

## 1. Introduction

*Zeugodacus cucurbitae* (Coquillett) poses a serious threat and has strong dispersal capabilities, infesting over 100 types of produce, such as *Momordica charantia*, *Cucurbita moschata*, and *Citrullus lanatus* [1,2]. The female adult uses its ovipositor to lay eggs inside the skin of fruit, and the larvae hatch and feed within the fruit, leading to their decay and causing a decrease in both the yield and quality of the crops [3,4,5]. Currently, the control and management of *Z. cucurbitae* primarily rely on chemical pesticides. However, research on biological control is continuously advancing, including methods such as parasitoids, bacteria, fungi, and nematodes [6]. The implementation of biological control will enhance the sustainability of agricultural ecosystems. Therefore, there is an urgent need for the investigation of novel biopesticides for the control and management of *Z. cucurbitae*.

Short-term high temperatures are considered a significant environmental factor affecting the development and reproduction of *Z. cucurbitae* [7]. With the ongoing climate warming, the incidence of extremely high temperatures is steadily increasing, and extensive research has been conducted on pests’ responses to high temperatures [8,9,10]. After subjecting *Zeugodacus tau* to a 12 h treatment at 34 °C, a notable increase in the mating rate was observed, while the lowest egg number and hatching rates were observed following treatment at 40 °C, indicating that high-temperature environments have an impact on pests’ reproductive behavior [11]. The optimal temperature range for *Z. cucurbitae*’s development lies between 23 and 30 °C, and the eggs, larvae, and pupae are unable to develop at the high temperature of 38 °C [12]. Among the various life stages of *Z. cucurbitae*, the adult stage exhibits the highest tolerance to elevated temperatures. The sub-lethal high temperature range for adults is observed between 41 and 47 °C, with temperatures above 51 °C resulting in fatality, and female adults demonstrate greater heat resistance than males [7,12]. In natural conditions, pests typically encounter short-term and fluctuating extreme high temperatures, as opposed to long-term and constant high temperatures [13,14]. By subjecting *Z. cucurbitae* adults to temperatures of 25, 33, 37, and 41 °C for 1 h, and recording indicators such as the egg number, the biological effects of short-term high temperatures on *Z. cucurbitae* reproduction were elucidated. It was observed that short-term high temperatures led to a decrease in the adult survival rate, a shortened lifespan, and a reduction in the egg-laying capacity of female adults with increasing temperatures. Short-term high temperatures significantly stimulate *Z. cucurbitae*’s mating, oviposition, and thermotactic behavior [15,16]. Moreover, short-term high temperatures can impact consecutive generations of *Z. cucurbitae*, resulting in decreased egg hatching rates, pupation rates, and eclosion rates in the subsequent generation. Identifying the effects of short-term high temperatures on *Z. cucurbitae*’s reproduction and lifespan is critical for the formulation of scientifically grounded strategies for *Z. cucurbitae* control during high-temperature seasons.

Vitellogenin (Vg) is a large-molecular protein specifically found in the bloodstreams of sexually mature non-mammalian ovipara. It serves as the precursor to vitellin (Vn), which is present in nearly all oviparous animals [17]. In insects, the Vg protein is a glycolipophosphoprotein of considerable size, composed of approximately 84% amino acids, 16% lipids, and 4% carbohydrates. Most insect Vg proteins consist of a large subunit (>150 kDa) and a small subunit (<65 kDa) and are formed through provitellogenin from enzymatic cleavage within the fat body [18,19]. Within the insect ovary, Vg is internalized by surrounding oocytes through vitellogenin receptor (VgR)-mediated endocytosis and further transformed into vitellin [20]. This process is vital for vitellogenesis and oocyte development. The primary function of vitellogenin is to serve as an energy source for insect oviposition and self-growth, encompassing various nutrients, such as amino acids, carbohydrates, lipids, phosphates, vitamins, and sulfates [18]. Intriguingly, vitellogenin in insects is a multifunctional protein. Besides providing nourishment for embryonic development, it also shows resistance to stress and oxidative damage, adaptation to different climates, ovarian activation, lifespan regulation, and wing polymorphism [21,22,23]. Furthermore, in social insects, Vg plays a crucial role in regulating the social division of labor and hierarchy [24]. The number of Vg isoforms varies among different insect species, with two Vgs found in *Leucophaea maderae* and *Riptortus clavatus*, three Vgs in *Drosophila melanogaster* and *Bactrocera dorsalis*, and four Vgs in *Solenopsis Invicta* [25,26,27,28,29].

Vg plays an essential role in the egg and ovarian development of adult insects. The RNA-interference-mediated inhibition of the *Vg* gene in *Romalea microptera* suppressed ovarian growth [30]. In *Anthonomus grandis*, *Vg* gene knockdown resulted in eggs losing viability [31]. Silencing the *ClVg* gene led to ovarian atrophy and reduced ovulation, directly inhibiting the reproduction of *Cimex lectularius* [32]. The RNA-interference-mediated knockdown of the *Vg* genes in *Z. cucurbitae* delayed ovarian development, indicating a close association between the *Vg* gene and adult reproduction [33].

The expression of *Vg* and *VgR* in *Z. cucurbitae* is influenced by short-term high temperatures. The exposure of the adults to short-term high temperatures of 25, 33, 37, 41, and 45 °C for 1 h revealed the differential expression of the *Vg* and *VgR* genes at both the transcriptional and protein levels using high-throughput sequencing techniques [34]. Against the background of short-term high temperatures, the RNA interference of the *ZcVgR* gene resulted in decreased expression and a 95.2% reduction in the egg number compared to the blank control, effectively suppressing the reproductive capacity of female adults, indicating the involvement of the *ZcVgR* gene in the regulation of female reproduction. Furthermore, the relative expression of the *ZcVg3* gene decreased after 24 h interference with the *ZcVgR* gene, suggesting that silencing the *ZcVgR* gene may lead to a decrease in vitellogenin-3 [35,36]. In this study, *ZcVg3* was used as the target gene. RNA interference was performed by injecting siRNA into *Z. cucurbitae* exposed at 45 °C for 1 h, and treatment at 25 °C for 1 h was used as the control. The expression of *ZcVg3* was measured using RT-qPCR to investigate the regulation of the *ZcVg3* gene in terms of female reproductive capacity in short-term high-temperature conditions, as well as the synergistic relationship between *ZcVgs* and *ZcVgR*. The results of this study reveal the potential of *ZcVg3* as a control target in *Z. cucurbitae* and provide a theoretical and practical reference for its prevention and control in high-temperature conditions.

## 2. Materials and Methods

### 2.1. Insect Rearing

The insect specimens used in this experiment were collected from bitter gourd cultivation sites in Nada Town, Danzhou City, Hainan Province, China (109°29′ E, 19°30′ N) and subsequently brought back to the laboratory for further rearing for many generations. The feed for both larvae and adults was prepared based on prescribed formulations [16]. Sodium benzoate and liquid nitrogen were procured from Hainan Qingfeng Biotechnology Co., Ltd. (Sanya, China). The indoor environment maintained an average temperature of 25 ± 1 °C and relative humidity of 70 ± 5% and followed a photoperiod of 14L:10D.

### 2.2. Design and Preparation of siRNA

Based on the conserved cDNA sequence of the *ZcVg3* gene, a 19-nucleotide siRNA was designed. The siRNA was synthesized by TsingkeBiotech Co., Ltd. (Beijing, China). The positive strand of the siRNA was 5′-GGAUCACACCAGAUUUGAA-3′, and the antisense strand was 5′-UUCAAAUCUGGUGUGAUCC-3′. The 21-nucleotide siRNA was designed based on the conserved cDNA sequence of the ZcVgR gene, with a positive strand of 5′-CGAUGUCGAGGAUGUGUUATT-3′ and an antisense strand of 5′-UAACACAUCCUCGACAUCGTT-3′. The negative control (NC) used was a commercially available siRNA that did not exhibit homology to the target cells and did not induce RNAi effects in any treatment. The NC siRNA contained the positive strand 5′-GGUUCUCCGAACGUGUCACGU-3′ and the antisense strand 5′-ACGUGACGUUCGGAGAACC-3′.

### 2.3. Injection of siRNA

The siRNA powder (2.5 nmoL) was dissolved in 125 μL of Tris–EDTA solution to prepare a 20 μM siRNA solution. Shortly after eclosion, adults were subjected to a high-temperature treatment at 45 °C for 1 h in a climate chamber (Hangzhou Qianjiang Instrument Equipment Co., Ltd., Hangzhou, China), with a control treatment at 25 °C for 1 h. After the treatment, the adults were maintained at room temperature (25 ± 1 °C). The injection site for female adults was selected as the second to third abdominal segments of the abdominal backplane. After 5 days of rearing, 1.25 μg (4.5 μL) of siRNA and the NC were injected using a pneumatic microinjector (IM-11-2, NARISHIGE, Setagaya, Japan). Control groups for the NC (received ineffective dsRNA), injury (underwent injection procedures but did not receive dsRNA), and CK (untreated control group) were set.

### 2.4. qRT-PCR Analysis

After injection, 20 female adults from the siRNA group, NC group, injury group, and CK group were placed in individual cages. Each cage contained as many males (without any treatment) as females for mating, and they were provided with artificial adult feed, water, and pumpkin slices for egg laying. The cages were maintained at 25 ± 1 °C. At 24, 48, and 72 h post-injection, 15 female adults were sampled from each group (siRNA, NC, injury, and CK). Five female adults were considered as one replicate, with three replicates per group. The adults were rapidly frozen using liquid nitrogen and stored in an ultra-low-temperature refrigerator.

The total RNA was extracted using the Animal Tissue/Cell Total RNA Extraction Kit (RNAprep FastPure, Qingke Biotechnology, Beijing, China). The extracted total RNA was detected by agarose gel electrophoresis (1.5% agarose gel, 1× TAE electrophoresis buffer) and absorbance detection. Subsequently, cDNA synthesis was performed using a reverse transcriptase kit (Goldenstar RT6 cDNA Synthesis Max, Qingke Biotechnology, China), according to the provided instructions. The cDNA product from reverse transcription was diluted three times and used as a template for qPCR. The agarose gel electrophoresis displayed bands of the expected sizes, without stray bands. The fusion curve analysis of the quantitative real-time PCR presented only a single peak, indicating high primer specificity and reliable results. The *succinate dehydrogenase flavoprotein* gene was chosen as the housekeeping gene based on preliminary screening [35]. The primers used in this experiment are listed in Table 1. The reaction mixture contained 1.0 μL of the cDNA template, 10.0 μL of 2× T5 Fast qPCR Mix (SYBR Green I), 1 μL of the upstream primer, 1 μL of the downstream primer, and 7.0 μL of ddH_2_O. The reaction procedure involved predenaturation at 95 °C for 2 min, denaturation at 95 °C for 15 s, annealing at 60 °C for 15 s, and extension at 72 °C for 20 s. A total of 41 cycles were performed, with three replicates per sample. Relative quantitative analysis was selected, and the original data were derived after the reaction. The C_T_ values of the internal reference gene and the target gene were determined using the 2^−ΔΔCT^ method [37], revealing the multiple relationships in the expression patterns of the samples. The mean value of the ΔC_T_ relative center data was taken as the control group data sample, i.e., the *VgR* gene was detected at 25 °C–negative control–24 h for the relative quantitative analysis.

### 2.5. Western Blot

At 24, 48, and 72 h after the siRNA injection of the *ZcVgR* gene, 15 adult females were extracted from the siRNA and CK. There were 5 female adults in 1 replicate and 3 replicates per group. The adult mice were quickly frozen with liquid nitrogen and sent to AtaGenix Laboratories Co., Ltd. (Wuhan), Wuhan, China. The protein expression of the *ZcVg3* gene was detected by Western blot.

The supernatants of the protein after dialysis were separated using 10% sodium dodecyl sulfate–polyacrylamide gel electrophoresis (SDS-PAGE). The gel images were captured using the ChemiDoc XRS^+^ system (Bio-Rad). Subsequently, the proteins in the gel were transferred to polyvinylidene fluoride (PVDF) membranes. The PVDF membranes were blocked with 5% milk for 1 h. Then, the solution was discarded and the membranes were rinsed with Tris-buffered saline with tween (TBST) for 2 min. 

The primary antibody was bound to the target protein and incubated overnight. Next, the membranes were again rinsed three times with TBST for 10 min each time. The PVDF membranes were placed into a secondary antibody labeled with horseradish peroxidase diluted with 5% milk and incubated at room temperature for 1 h. The membranes were again rinsed with TBST three times. The bands in each membrane were observed with the ChemiDoc XRS^+^ system.

### 2.6. Effect of Silencing ZcVg3 Gene on Reproduction and Lifespan of Z. cucurbitae (Coquillett)

Five females from each of the siRNA, NC, injury, and CK groups were placed in individual cages with 5 males each (without any treatment) for mating. This was a biological replicate and 6 replicates were set. The adults were provided with artificial adult feed, water, and pumpkin slices for egg laying and were maintained at 25 ± 1 °C. The following parameters were recorded: egg number, oviposition duration (d), hatchability of eggs (%), and lifespan (d).

### 2.7. Effect of Silencing ZcVg3 Gene on Ovarian Development of Z. cucurbitae (Coquillett)

For the siRNA, NC, injury, and CK groups, 5 female adults were placed in individual cages. Five male adults were paired as one replicate, setting six replicates per group. The adults were provided with artificial adult feed, water, and pumpkin slices for egg laying and were maintained at 25 ± 1 °C. Then, 11-, 12-, and 13-day-old female adults were dissected after injection, with 5 female adults dissected per treatment per day, and the ovaries were photographed and recorded.

### 2.8. Statistics

The data were analyzed using Excel (2021 version, Microsoft, Redmond, DC, USA) and SPSS (version 27.0, IBM, Armonk, NY, USA) through a completely randomized ANOVA and Tukey’s multiple comparisons. Proportional data were first transformed using the square root of the inverse sine and then analyzed via ANOVA. All values in the results are presented as the mean ± standard error.

## 3. Results

### 3.1. Silencing Efficiency of ZcVg3 Gene

**Figure 1:**
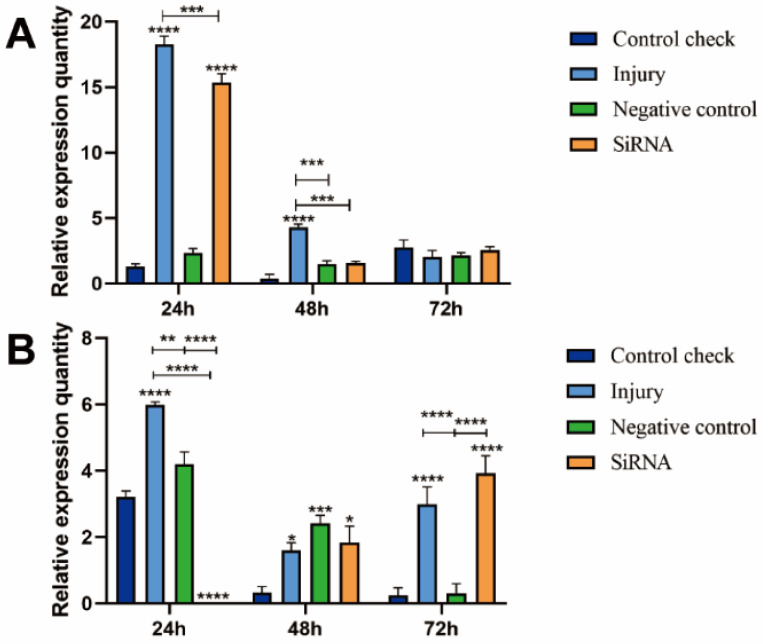
Silencing efficiency of *Vg3* gene in *Z. cucurbitae* (Coquillett). (**A**) Interfering target genes after 25 °C treatment for 1 h (*F*_(3, 24)_ = 205.7, *p* < 0.0001; *F*_(2, 24)_ = 425.2, *p* < 0.0001; *F*_(6, 24)_ = 144.2, *p* < 0.0001). (**B**) Interfering target genes after 45 °C treatment for 1 h (*F*_(3, 24)_ = 26.05, *p* < 0.0001; *F*_(2, 24)_ = 35.23, *p* < 0.0001; *F*_(6, 24)_ = 37.30, *p* < 0.0001). All values in the figure are presented as the mean ± standard error. *, **, ***, and **** above the histograms indicate the values that differ significantly between treatments, *p* ≤ 0.05, *p* ≤ 0.01, *p* ≤ 0.001, and *p* ≤ 0.0001, respectively.

The results showed that the siRNA group had a significant reduction in the expression of the *ZcVg3* gene at 24 and 48 h, respectively, compared to the injury group at 25 °C (Figure 1A). The siRNA group maintained low expression levels at 48 and 72 h, with no significant differences among the treatments at 72 h. At 45 °C, the relative expression quantities of the CK, NC, and injury groups reached their peaks at 24 h, being significantly higher than the siRNA group (Figure 1B). This indicated the valid silencing of the *ZcVg3* gene expression.

### 3.2. Effect of Silencing ZcVgR Gene on Vitellogenin-3 Expression

**Figure 2:**
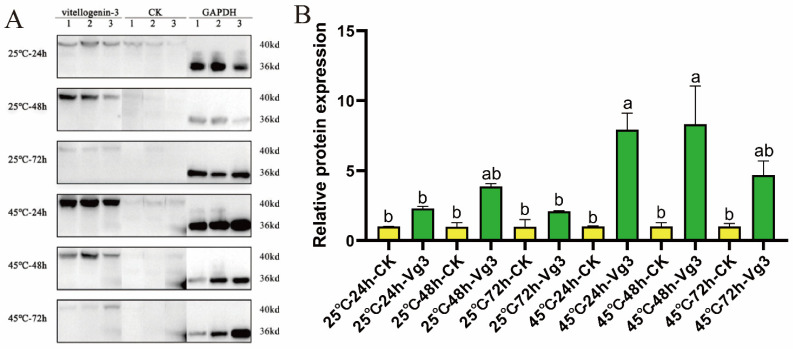
Western blot analysis of *Vg3* gene after interference with *VgR* gene. (**A**) Western blot strip diagram. Three replicates were performed for each treatment (File S1). (**B**) Western blot analysis of gray scale. All values in the figure are presented as the mean ± standard error. The letters above the bars indicate significant differences (*p* ≤ 0.05).

The Western blot results revealed that vitellogenin-3 had bands around 40 kDa (Figure 2A). After injecting the siRNA with the *ZcVgR* gene, the expression of vitellogenin-3 was observed to be higher than in CK after 24 and 48 h (Figure 2B). This suggests that the silencing of the *ZcVgR* gene can increase the expression of the *ZcVg3* gene at the protein level. This result is consistent with the observation that the transcriptional level of the *ZcVg3* gene also increases after silencing the *ZcVgR* gene. Moreover, the expression of vitellogenin-3 was higher at 45 °C than at 25 °C after 24 h of injection, suggesting that short-term high temperatures may stimulate its expression.

### 3.3. Effect of Silencing ZcVg3 Gene on ZcVgR Gene Expression

**Figure 3:**
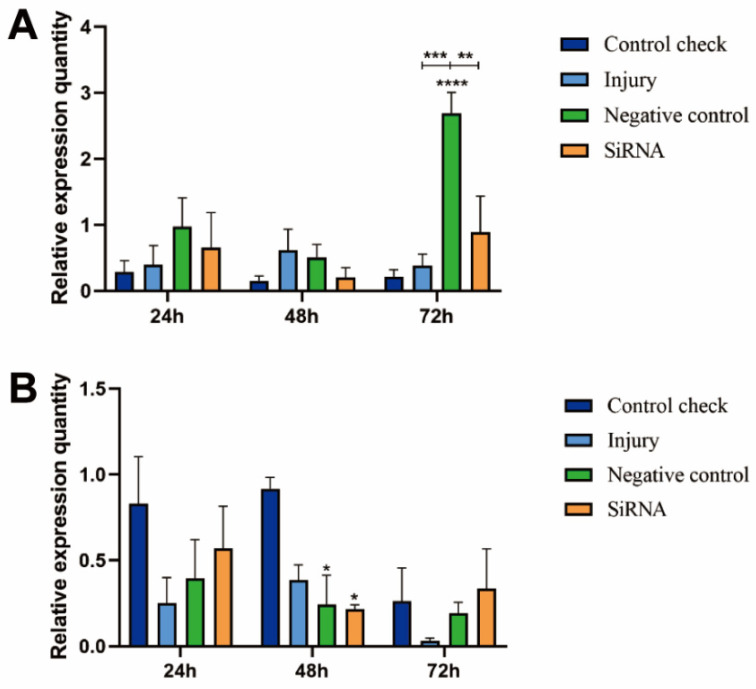
Effect of silencing *ZcVg3* gene on *ZcVgR* gene expression in *Z. cucurbitae* (Coquillett). (**A**) Interfering target genes after 25 °C treatment for 1 h (*F*_(3, 24)_ = 7.787, *p* = 0.0008; *F*_(2, 24)_ = 4.795, *p* = 0.0177; *F*_(6, 24)_ = 3.323, *p* = 0.0159). (**B**) Interfering target genes after 45 °C treatment for 1 h (*F*_(3, 24)_ = 4.163, *p* = 0.0165; *F*_(2, 24)_ = 3.568, *p* = 0.0440; *F*_(6, 24)_ = 1.153, *p* = 0.3632). All values in the figure are presented as the mean ± standard error. *, **, ***, and **** above the histograms indicate the values that differ significantly between treatments, *p* ≤ 0.05, *p* ≤ 0.01, *p* ≤ 0.001, and *p* ≤ 0.0001, respectively.

The results showed that the relative expression levels of the CK and injury groups were lower at 25 °C (Figure 3A). The siRNA group showed a pattern of downregulation followed by upregulation, with the highest level of expression observed at 72 h. At 45 °C, the relative expression levels of the CK group and injury group first increased and then decreased (Figure 3B). The siRNA group still showed a trend of downregulation and upregulation.

### 3.4. Effect of Silencing ZcVg3 Gene on ZcVg1 Gene Expression

**Figure 4:**
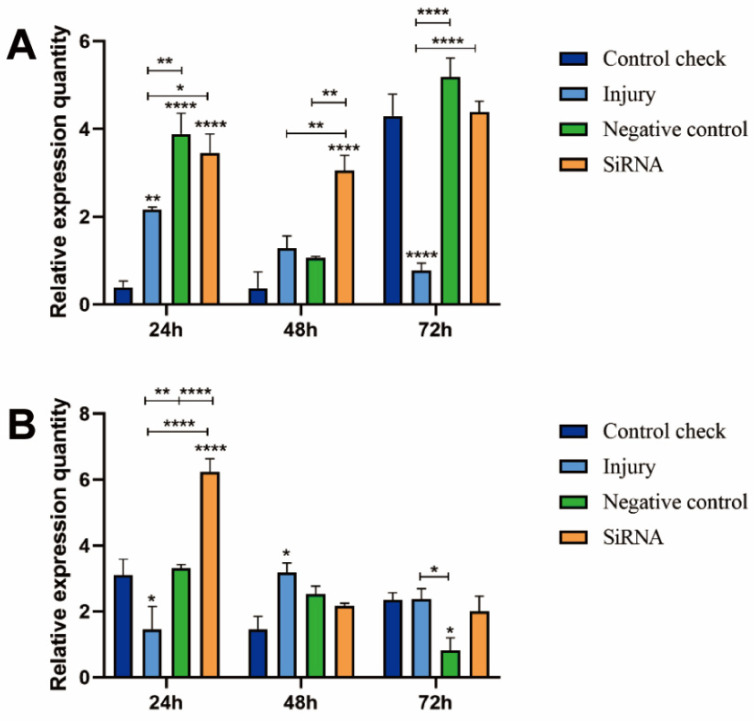
Effect of silencing *ZcVg3* gene on *ZcVg1* gene expression in *Z. cucurbitae* (Coquillett). (**A**) Interfering target genes after 25 °C treatment for 1 h (*F*_(3, 24)_ = 36.28, *p* < 0.0001; *F*_(2, 24)_ = 45.82, *p* < 0.0001; *F*_(6, 24)_ = 17.31, *p* < 0.0001). (**B**) Interfering target genes after 45 °C treatment for 1 h (*F*_(3, 24)_ = 7.582, *p* = 0.0010; *F*_(2, 24)_ = 20.45, *p* < 0.0001; *F*_(6, 24)_ = 14.01, *p* = 0.0967). All values in the figure are presented as the mean ± standard error. *, **, and **** above the histograms indicate the values that differ significantly between treatments, *p* ≤ 0.05, *p* ≤ 0.01, and *p* ≤ 0.0001, respectively.

The results indicated the significantly higher relative expression of the siRNA group at 25 °C than that in the CK, injured, and NC groups after 48 h (Figure 4A). At 24 h, the siRNA group had significantly higher relative expression at 45 °C compared to the CK, injured, and NC groups (Figure 4B). This indicates that the silencing of the *ZcVg3* gene leads to the upregulation of the expression level of the *ZcVg1* gene.

### 3.5. Effect of Silencing ZcVg3 Gene on ZcVg2 Gene Expression

**Figure 5:**
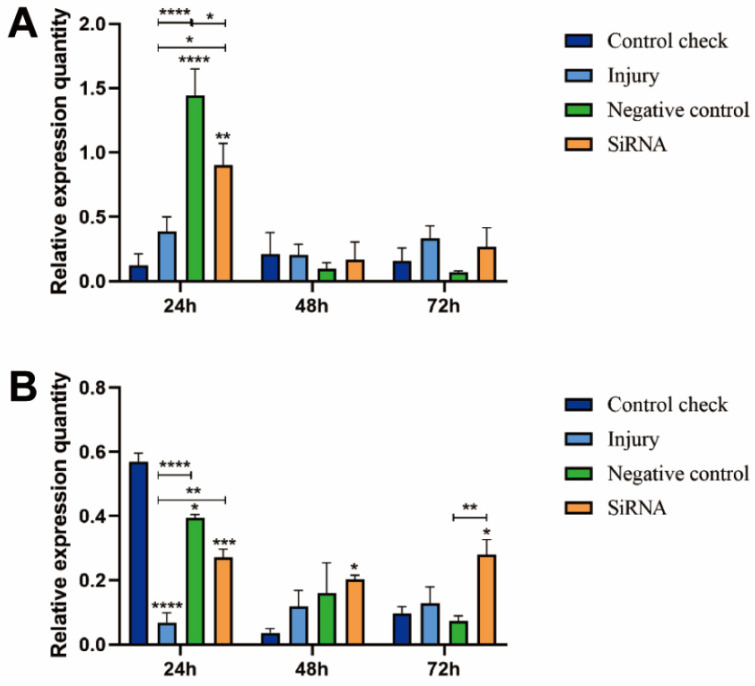
Effect of silencing *ZcVg3* gene on *ZcVg2* gene expression in *Z. cucurbitae* (Coquillett). (**A**) Interfering target genes after 25 °C treatment for 1 h (*F*_(3, 24)_ = 5.081, *p* = 0.0073; *F*_(2, 24)_ = 23.60, *p* < 0.0001; *F*_(6, 24)_ = 8.822, *p* < 0.0001). (**B**) Interfering target genes after 45 °C treatment for 1 h (*F*_(3, 24)_ = 7.929, *p* = 0.0008; *F*_(2, 24)_ = 29.46, *p* < 0.0001; *F*_(6, 24)_ = 13.93, *p* < 0.0001). All values in the figure are presented as the mean ± standard error. *, **, ***, and **** above the histograms indicate the values that differ significantly between treatments, *p* ≤ 0.05, *p* ≤ 0.01, *p* ≤ 0.001, and *p* ≤ 0.0001, respectively.

The results showed that, at 25 °C, the relative expression of the siRNA group at 24 h was significantly higher than that of the CK and injured groups (Figure 5A). However, the relative expression level decreased at 48 and 72 h, with no significant differences observed among the treatments. At 45 °C, the relative expression of the siRNA group was significantly higher after 72 h compared to the CK and NC groups (Figure 5B). This suggests that the *ZcVg3* gene’s silencing may lead to the upregulation of the expression level of the *ZcVg2* gene in short-term high-temperature conditions.

### 3.6. Effect of Silencing ZcVg3 Gene on Reproduction and Lifespan of Z. cucurbitae (Coquillett)

**Figure 6:**
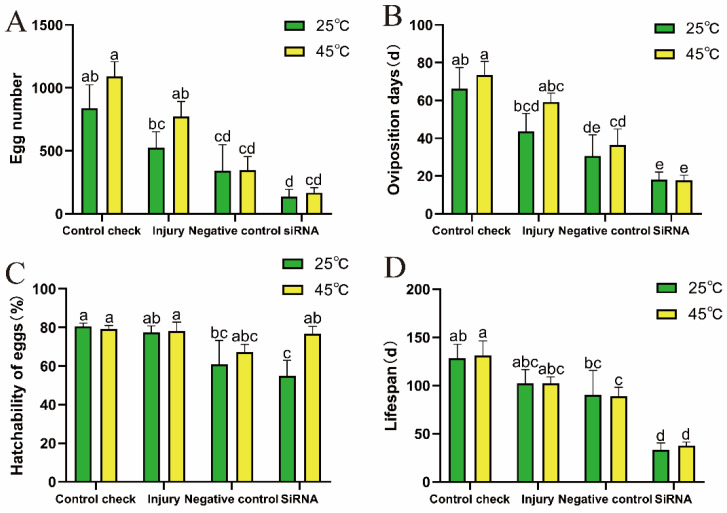
Effect of silencing *ZcVg3* gene on reproduction and lifespan of *Z. cucurbitae* (Coquillett). (**A**) Egg number (25 °C, *F* = 3.715, *p* = 0.0284; 45 °C, *F* = 16.960, *p* = 0.0001). (**B**) Oviposition days (25 °C, *F* = 4.709, *p* = 0.0121; 45 °C, *F* = 15.540, *p* = 0.0001). (**C**) Hatchability of eggs (25 °C, *F* = 2.652, *p* = 0.0766; 45 °C, *F* = 2.226, *p* = 0.1165). (**D**) Lifespan (25 °C, *F* = 5.784, *p* = 0.0051; 45 °C, *F* = 15.90, *p* < 0.0001). All values in the figure are presented as the mean ± standard error. The letters above the bars indicate significant differences (*p* ≤ 0.05).

Following siRNA injection in 5-day-old female adults, the egg number (Figure 6A), oviposition days (Figure 6B), and hatchability of eggs (Figure 6C) were recorded. The results demonstrate that, at 25 °C, the siRNA treatment exhibited the lowest total egg number and oviposition days, with values of 136 and 18.0 d, respectively. These values were significantly reduced compared to CK (840 and 66.2 d), showing reductions of approximately 83.8% and 72.8%, respectively. At 45 °C, the siRNA treatment resulted in the lowest total egg number and oviposition days, with values of 167 and 17.7 d, significantly lower than CK (1092 and 73.3 d), exhibiting reductions of approximately 84.7% and 75.9%, respectively. Interfering with the *ZcVg3* gene caused a decline in the reproductive capacity of the female adults, indicating the crucial role of the *ZcVg3* gene in the reproductive process of *Z. cucurbitae* in short-term high-temperature conditions.

At 25 °C, the siRNA treatment resulted in the shortest lifespan among the female adults, with a duration of 33 d (Figure 6D), significantly lower than in the other treatments. The female lifespan in the injury (102 d) and negative control (NC) groups (90 days) showed no significant differences compared with that of CK (128 d). At 45 °C, CK exhibited the longest lifespan among all treatments, lasting 131 d, while siRNA had the shortest lifespan, lasting 38 d, indicating that interfering with the *ZcVg3* gene can significantly reduce the lifespan of *Z. cucurbitae*.

### 3.7. Effect of Silencing ZcVg3 Gene on Ovarian Development of Z. cucurbitae (Coquillett)

**Figure 7:**
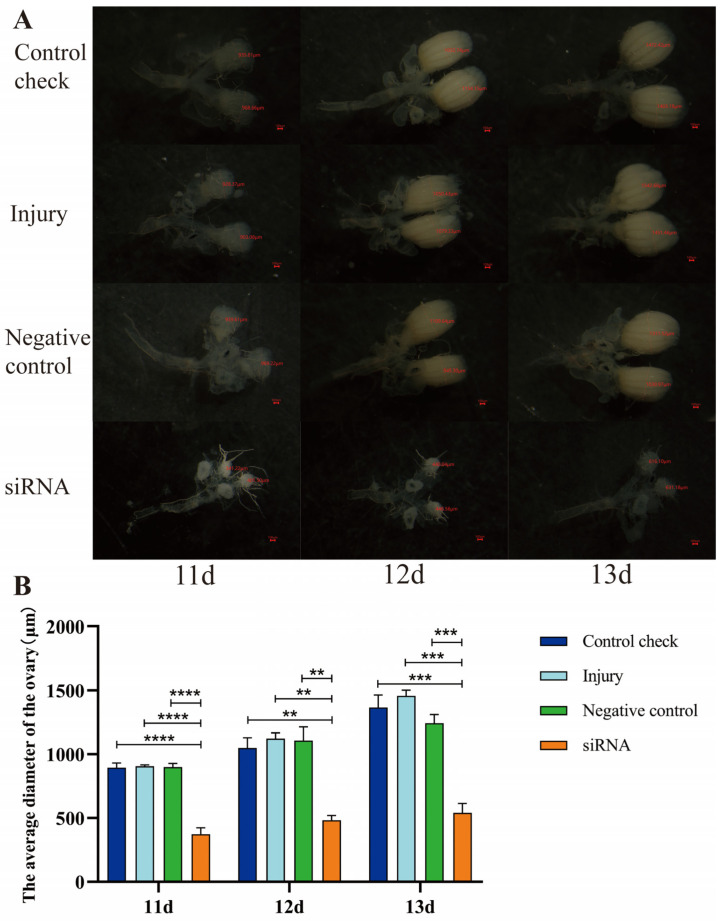
Effect of silencing *ZcVg3* gene on ovarian development of *Z. cucurbitae* (Coquillett) after 25 °C treatment for 1 h. (**A**) Rate of ovarian development. The red line denotes the diameter of the ovary (μm). The red scale is 100 μm. (**B**) Average diameter of ovary (*F*_(3, 24)_ = 80.42, *p* < 0.0001; *F*_(2, 24)_ = 36.95, *p* < 0.0001; *F*_(6, 24)_ = 2.058, *p* = 0.0967). All values in the figure are presented as the mean ± standard error. **, ***, and **** above the histograms indicate the values that differ significantly between treatments, *p* ≤ 0.01, *p* ≤ 0.001, and *p* ≤ 0.0001, respectively.

**Figure 8:**
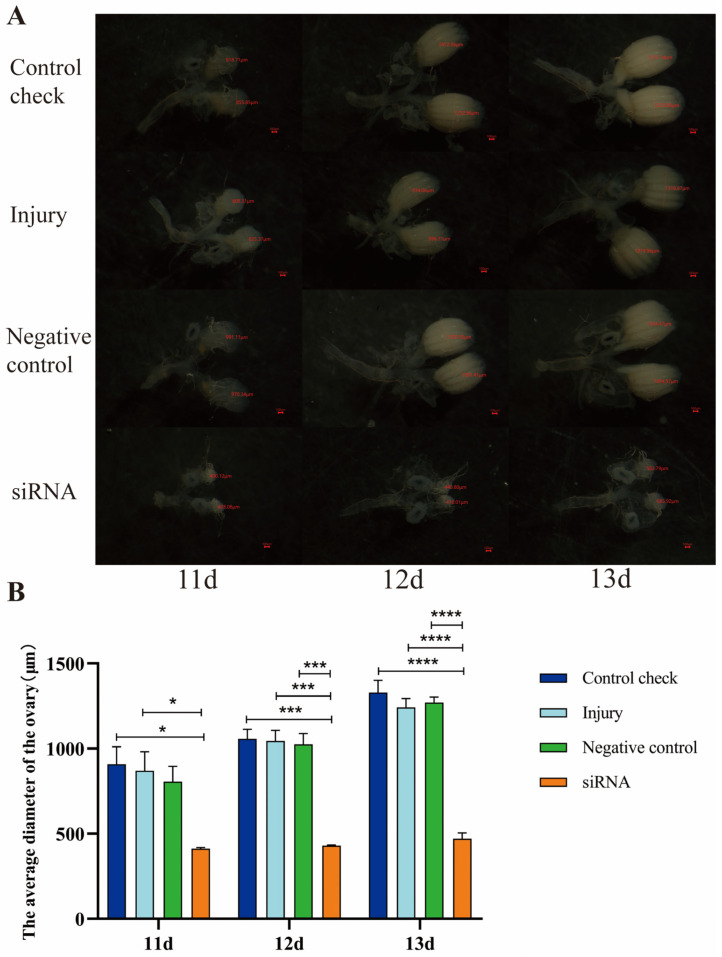
Effect of silencing *ZcVg3* gene on ovarian development of *Z. cucurbitae* (Coquillett) after 45 °C treatment for 1 h. (**A**) Rate of ovarian development. The red line denotes the diameter of the ovary (μm). The red scale is 100 μm. (**B**) Average diameter of ovary (*F*_(3, 24)_ = 66.18, *p* < 0.0001; *F*_(2, 24)_ = 24.55, *p* <0.0001; *F*_(6, 24)_ = 1.959, *p* = 0.1119). All values in the figure are presented as the mean ± standard error. *, ***, and **** above the histograms indicate the values that differ significantly between treatments, *p* ≤ 0.05, *p* ≤ 0.001, and *p* ≤ 0.0001, respectively.

Following siRNA injection in 5-day-old female adults, and after five days, 11-, 12-, and 13-day-old female adults were dissected. The results indicate that, under the 25 °C (Figure 7) and 45 °C treatments for 1 h (Figure 8), the ovarian diameter of the siRNA group at 11, 12, and 13 d was significantly smaller compared to that of the CK, NC, and injury groups. Furthermore, the ovarian development rate of the siRNA group was significantly slower than that in the other treatments, providing evidence that silencing the *ZcVg3* gene inhibits ovarian development in *Z. cucurbitae*.

## 4. Discussion

The production and accumulation of vitellogenin are pivotal stages in insect reproduction. In this study, the suppression of *ZcVg3* expression markedly reduced the egg number and oviposition days in female adults. Compared to individuals not injected with siRNA, siRNA-injected female adults revealed shorter ovarian diameters and slower ovarian development rates. These findings manifest the considerable role of *ZcVg3* in the reproductive process of *Z. cucurbitae*. Chen et al. [33] observed a similar effect on ovarian development with the knockdown of the *ZcVgs* gene by injecting dsRNA, aligning with the results of this study and highlighting the essentiality of *ZcVgs* gene expression for ovarian development. The egg number of *Z. cucurbitae* may also be correlated with internal nutrient substances. Subjecting adults to a 24 h starvation treatment resulted in the downregulation of *ZcVgs* expression, which was subsequently restored upon nutrient supplementation [33,38]. This underscores the importance of nutrients in the process of insect vitellogenesis. In this study, both the NC and injury groups showed slightly lower total egg numbers compared to CK, possibly due to the usage of their own nutrients for wound healing among the female adults. This reduction in the available nutrients required for egg development subsequently led to a decrease in the overall egg number. Further research is needed to investigate the relationship between internal nutrient substances and the genes involved in the process of vitellin formation in insects.

The silencing of *Vg* can also result in a decreased reproductive capacity and abnormal ovarian development in other insects. The downregulation of the *Vg* gene in *Panonychus citri* resulted in a 48% reduction in egg number, with 48.14% of females being unable to oviposit [39]. Silencing the *Vg* gene in *Rhynchophorus ferrugineus* caused a decrease in egg number, a delayed oviposition period, a significantly reduced egg hatching rate, and ovarian atrophy [40]. These studies collectively indicate that the silencing of the *Vg* gene significantly diminishes the reproductive capacity of female insects and inhibits ovarian development. Vg is an indispensable protein in insect reproduction.

The expression of the *Vgs* and *VgR* genes in insects is mutually influenced. In *Nilaparvata lugens*, interference with *NlVgR* led to a decrease in the *NlVg* protein in the ovaries and the accumulation of the *NlVg* protein in the hemolymph [41]. Western blot analysis showed the increased expression of the *ZcVg3* protein after silencing the *ZcVgR* gene, which was consistent with *ZcVgR* transcript detection [35]. The expression of the *ZcVgR* gene at the transcriptional level decreased first and then increased after the *ZcVg3* gene was silenced. This finding suggests that *Vg3* may be the primary ligand for *VgR*. In this study, the silencing of *ZcVg3* resulted in the upregulation of *ZcVg1* and *ZcVg2* gene expression, indicating that the expression of *Vgs* may involve their interaction. Silencing *Vg-2* in *Haemaphysalis longicornis* caused a decrease in *Vg-1* gene expression [42]. The expression of *Vgs* genes in insects exhibits a synergistic effect, although the precise mechanisms are still to be elucidated.

Insects possess multiple types of *Vgs*, which exhibit structural and functional disparities. In *N. lugens*, *Vg* and *Vg-like2* are predominantly expressed in female adults, while *Vg-like1* is expressed at all stages in both males and females. Silencing the *Vg-like1* gene could have an impact on egg development [43]. The interplay of the *Vg* and *Vg-like* genes influences the growth and development of insects, rendering them potential targets for pest management strategies based on RNA interference. Further research is needed to delve into their specific functions.

The expression of the *Vg3* gene in *Z. cucurbitae* is associated with its lifespan. After subjecting female adults to 25 and 45 °C for 1 h, their lifespan decreased by 74 and 71%, respectively, indicating that silencing the *ZcVg3* gene shortened the lifespan of these female adults. The juvenile hormone (JH) serves as a primary hormone regulating vitellogenin production and plays a role in the reproductive maturation processes of adult insects, promoting the growth and development of the male accessory glands and female ovaries [44,45,46]. The removal of the pharyngeal body in new *D. melanogaster* reduced JH, generating an extended lifespan, suggesting a negative correlation between JH and longevity [47]. Based on this, it can be inferred that the interference with the *ZcVg3* gene in this study resulted in a reduction in vitellogenin-3, leading to the upregulation of JH and subsequently reducing the lifespan of *Z. cucurbitae*. The decreased lifespan observed in the silenced group, irrespective of temperature exposure, may have been due to the crucial role of the silenced gene in maintaining normal physiological function and stress responses in the insect. Silencing this gene likely compromises the insect’s ability to cope with stress. Further research is needed to investigate the molecular mechanisms underlying the impact of the *ZcVg3* gene on *Z. cucurbitae*’s lifespan.

## 5. Conclusions

In this study, the silencing effect of siRNA on the *ZcVg3* gene and its effects on the expression of the *ZcVg1*, *ZcVg2*, and *ZcVgR* genes, as well as the reproductive capacity and lifespan of female adult *Z. cucurbitae*, were investigated. The results showed that the expression of the *ZcVg3* gene was successfully silenced. This silencing effect had a significant effect on the expression of the *ZcVg1*, *ZcVg2*, and *ZcVgR* genes at different temperatures. Specifically, the silencing of the *ZcVg3* gene led to the upregulation of the *ZcVg1* and *ZcVg2* genes. These findings reveal the important role of the *ZcVg3* gene in regulating the expression of other related genes.

In addition, the study also found that *ZcVg3* gene silencing significantly reduced the reproductive capacity of female adults, including the number of eggs laid, oviposition days, and ovarian development. This effect was significant at both 25 and 45 °C, indicating that the *ZcVg3* gene plays a key role in the reproductive process of *Z. cucurbitae*. At the same time, the silencing of the *ZcVg3* gene also significantly shortened the lifespan of the female adults, which was verified in both experimental temperature conditions.

In summary, this study silenced the *ZcVg3* gene via siRNA injection technology and revealed the important role of this gene in regulating the expression of other related genes and affecting the reproductive ability and lifespan of *Z. cucurbitae*. These findings provide valuable experimental data and a theoretical basis for a further understanding of *Z. cucurbitae* and the development of new pest control strategies.

## Figures and Tables

**Table 1:** Primer sequence information for target gene and reference gene.

Primer Name	Primer Sequence (5′ to 3′)	Use of Primers
*Succinate dehydrogenase flavoprotein*-F	TTGATTTCAAAATAGGCGCAGTG	Reference gene amplification in qPCR
*Succinate dehydrogenase flavoprotein*-R	CGATGGTACACGCATAAGGC	
*Vitellogenin receptor*-F	TGCTTTCCCGGTTATCGCTT	Target gene amplification in qPCR
*Vitellogenin receptor*-R	AACGTAATCGGTTGCTCCGT	
*Vitellogenin-1*-F	TGCCACGTGACTTAATCGGT	
*Vitellogenin-1*-R	ATGCTGTGGCTAGAGGCCAT	
*Vitellogenin-2*-F	GCTTGTAATGAGTCGGTGGCG	
*Vitellogenin-2*-R	AGTGGCGCAAAGAAATGCCT	
*Vitellogenin-3*-F	TTTGCTCCTCAGCACTCTCA	
*Vitellogenin-3*-R	GCGCCATATTTGATCGGCA	

## Data Availability

The original contributions presented in the study are included in the article, further inquiries can be directed to the corresponding authors.

## Supplementary Materials

The following supporting information can be downloaded at: https://www.mdpi.com/article/10.3390/insects15070499/s1, File S1: Original Western blot figures.
