# Supplementary figures and images for: The ZcVg3 Gene Regulates the Reproduction and Lifespan of Female Zeugodacus cucurbitae (Coquillett) Mediated by Short-Term High Temperatures
Source: Insects. 2024 Jul 4;15(7):499. doi: 10.3390/insects15070499 (PMC11277402; doi:10.3390/insects15070499)

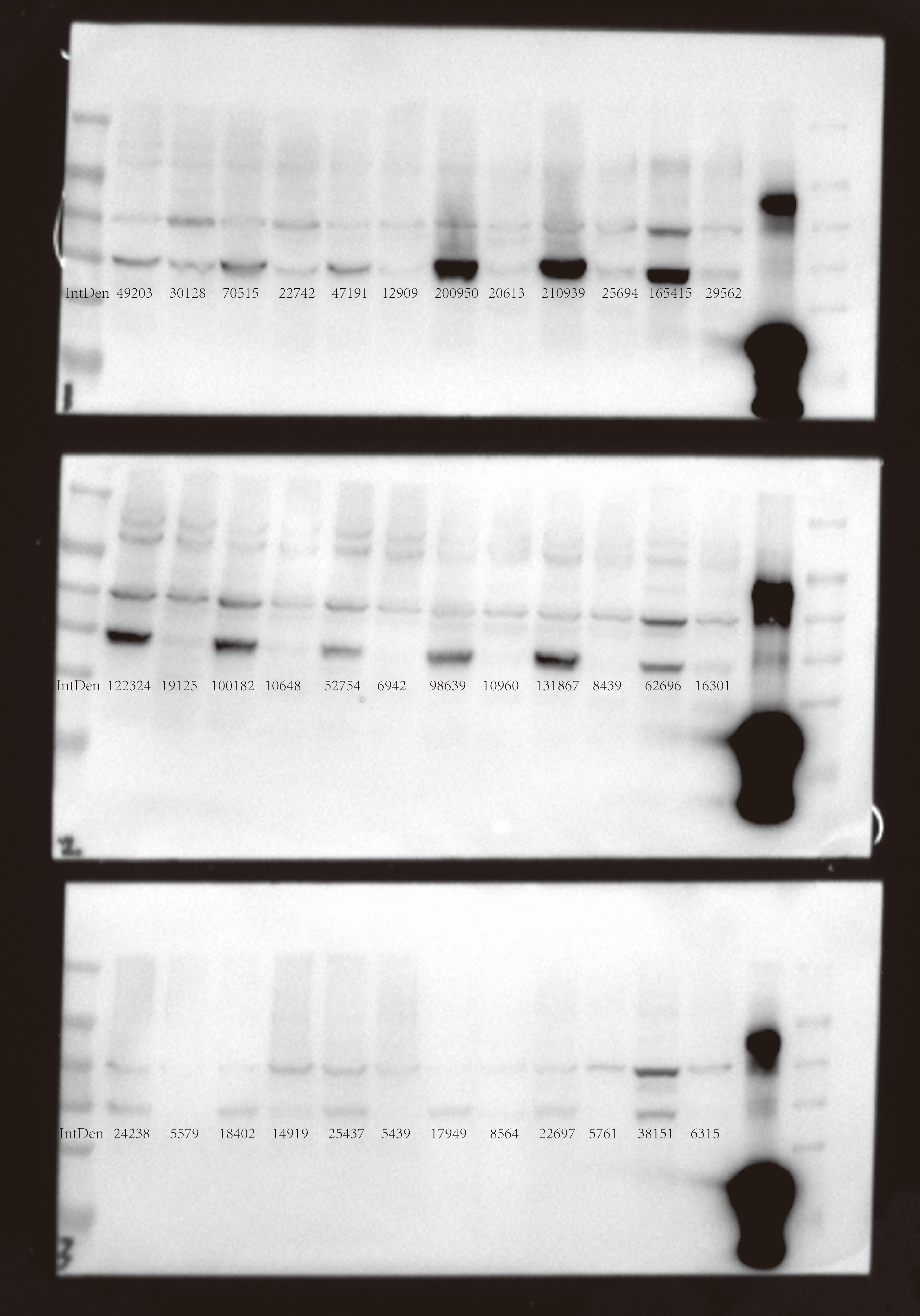

Supplement: Supplementary file 1 [file insects-15-00499-s001.zip › File S1/WB -Vg3.jpg]

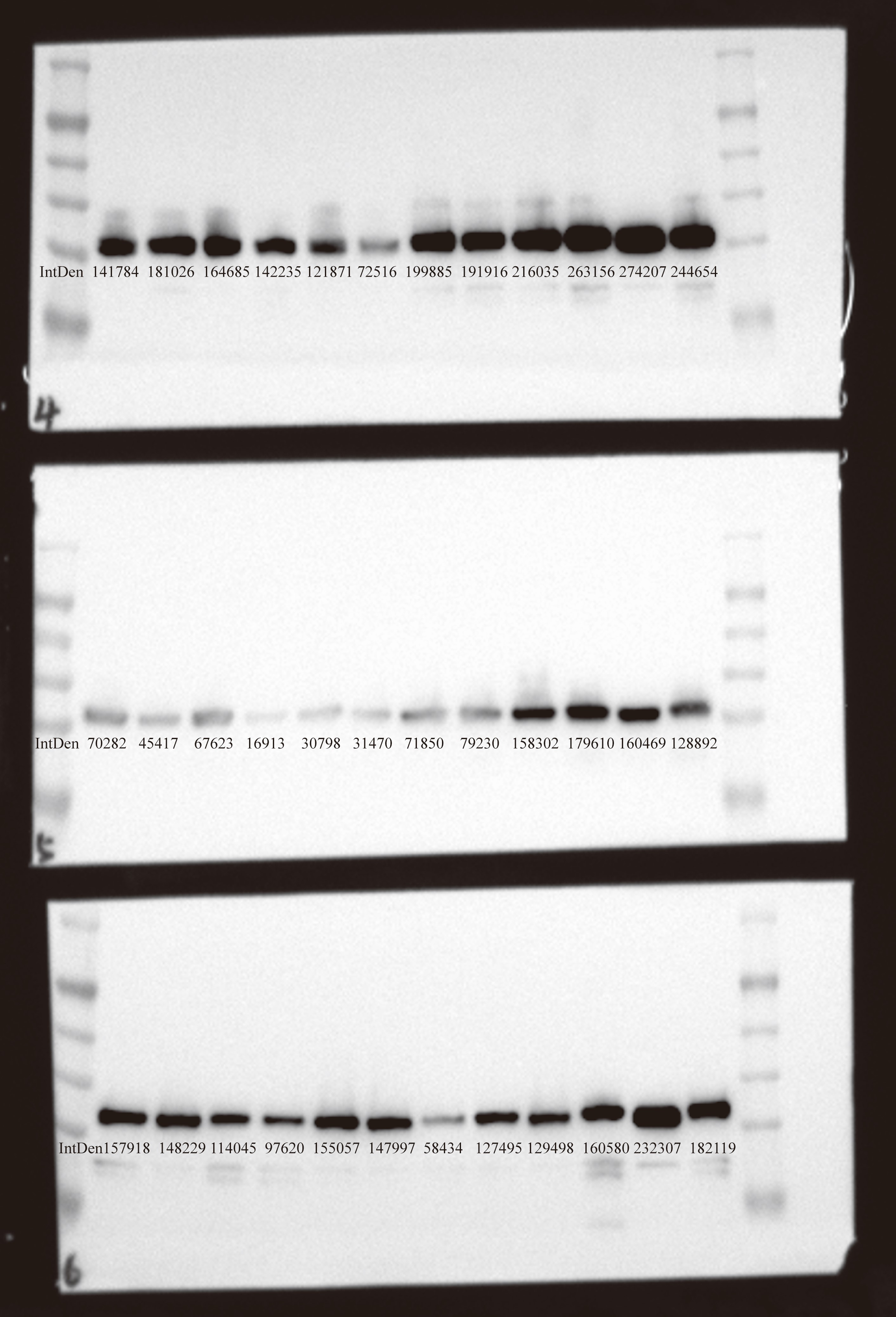

Supplement: Supplementary file 1 [file insects-15-00499-s001.zip › File S1/WB-GAPDH.jpg]
